# Factors affecting the umbilical artery Doppler reference values in the second and third trimesters

**DOI:** 10.1002/ijgo.70597

**Published:** 2025-10-17

**Authors:** Sophia Rahimi, John C. Kingdom, Arietta Vayenas, Vasilica Stratulat, Nir Melamed

**Affiliations:** ^1^ Temerty Faculty of Medicine University of Toronto Toronto Ontario Canada; ^2^ Division of Maternal‐Fetal Medicine, Department of Obstetrics and Gynaecology Sunnybrook Health Sciences Centre Toronto Ontario Canada; ^3^ Division of Maternal‐Fetal Medicine, Department of Obstetrics and Gynaecology Mount Sinai Hospital Toronto Ontario Canada

**Keywords:** chart, heterogeneity, reference, umbilical artery Doppler, variation

## Abstract

**Objective:**

To explore the contribution of selected methodological factors to the heterogeneity in published umbilical artery pulsatility index (UA‐PI) reference charts.

**Methods:**

Cross‐sectional study of uncomplicated singleton pregnancies that underwent assessment of UA Doppler at a single center. We explored the effects of the cohort characteristics (parity and estimated fetal weight [EFW] centile cut‐off) and the statistical modeling approach used to construct the UA‐PI centile reference charts.

**Results:**

25 069 UA‐PI measurements from 12 394 patients were analyzed. UA‐PI centile values decreased as the minimal EFW centile inclusion cut‐off of the study cohort increased. Interpretation of UA‐PI using charts constructed from fetuses with EFW > 25th or 50th centiles resulted in a higher proportion of examinations with UA‐PI > 95th centile compared with the chart based on fetuses with EFW⟩10th centile (11.5% and 13.2% versus 10.6% of the subgroup of small‐for‐gestational‐age fetuses, respectively, *P* < 0.001). In contrast, parity and the statistical method used to construct the chart had minimal impact on the UA‐PI centile reference charts. Considerable heterogeneity was identified among published UA‐PI reference charts.

**Conclusion:**

Variation in the distribution of EFW centiles across the populations used to construct the UA‐PI reference chart may contribute to the heterogeneity observed in published charts.

## 1 INTRODUCTION

Fetal growth restriction (FGR) is defined as the failure of a fetus to achieve its growth potential, most often due to varying degrees of underlying placental insufficiency.^1^ FGR is a common pregnancy complication and a leading cause of adverse perinatal outcomes, including stillbirth, asphyxia, and neonatal morbidity and mortality.^2^, ^3^

One of the key tools used to distinguish growth‐restricted fetuses from those constitutionally small for gestational age (SGA), as well as to guide antenatal surveillance and the timing of delivery, is umbilical artery (UA) Doppler. The importance of UA Doppler is illustrated by its inclusion in FGR diagnostic criteria^4^ and its recommended use by national and international FGR guidelines.^5^, ^6^, ^7^, ^8^, ^9^, ^10^, ^11^ However, the interpretation of UA Doppler in clinical practice is limited by substantial heterogeneity among published UA Doppler centile reference charts [12–20]. For example, a recent systematic review found that the proportion of SGA fetuses classified as growth‐restricted based on the presence of abnormal UA pulsatility index (UA‐PI) varied between 24.5% and 2.1%, depending on which UA‐PI centiles chart was used.^21^

One possible explanation for the heterogeneity across existing UA Doppler centiles charts is differences in their study populations. Factors such as maternal age, race, parity, and fetal size have each been shown to affect UA‐PI values.^15^, ^22^ Additionally, although most studies included only clinically low‐risk pregnancies to construct a UA‐PI standard reflecting the normal range of UA‐PI at each gestational week,^12^, ^13^, ^14^, ^16^, ^18^ others constructed UA‐PI reference charts that included pregnancies with maternal comorbidities and pregnancy complications such as pre‐eclampsia, FGR, and diabetes.^15^ Another potential explanation is the statistical methodology chosen to construct these charts.^18^, ^23^, ^24^ Currently, data on the impact of these factors (study population characteristics and the statistical approach) on the resultant UA‐PI centiles chart are lacking. A better understanding of the influence of these factors could identify important and readily addressed limitations affecting the application of UA Doppler in the diagnosis and management of suspected FGR.

In the current study, we aimed to explore the potential impact of selected factors (parity, the minimal estimated fetal weight [EFW] centile inclusion cut‐off, and the statistical method used to construct the chart) on the resulting UA‐PI centiles chart using a large contemporary population of healthy, uncomplicated singleton pregnancies. We subsequently constructed a new UA‐PI centiles chart based on the optimal combination of these factors and compared its performance with that of previously published charts.

## 2 MATERIALS AND METHODS

### 2.1 Study design and participants

We conducted a cross‐sectional study of uncomplicated singleton pregnancies that underwent sonographic assessment of UA Doppler and resulted in a live, term birth at a single tertiary referral center (Sunnybrook Health Sciences Centre, Toronto, ON, Canada) between January 2012 and December 2022. Pregnancies complicated by any of the following conditions were excluded: (1) maternal age less than 18 or more than 40 years; (2) preexisting diabetes mellitus or hypertension; (3) major structural or genetic abnormalities; (4) pregnancy complications, including gestational diabetes, gestational hypertension, or pre‐eclampsia; (5) gestational age at birth less than 37^+0^ weeks; (6) gestational age at the ultrasound examination less than 20^+0^ weeks or more than 42^+0^ weeks; or (7) ultrasound examinations without recorded gestational age or EFW. The study was approved by the Institutional Research Ethics Board #353–2014.

### 2.2 Data source

Cases were identified from the obstetrical ultrasound unit database. Ultrasound data included gestational age at the time of the ultrasound examination, EFW, and UA‐PI. Throughout the study period, our ultrasound unit protocol included the routine measurement of UA‐PI in all patients referred for fetal growth or biophysical profile assessment. Information on maternal characteristics, medical and obstetrical history, validation of estimated due date, pregnancy complications, gestational age at birth, birth weight, and neonatal outcomes was obtained from patients' electronic medical records.

### 2.3 Umbilical artery Doppler measurements

All sonographic examinations were performed by certified sonographers trained in obstetrical ultrasound and were reviewed by maternal‐fetal medicine specialists or radiologists with expertise in obstetrical ultrasound. Images were obtained using Voluson 730/830/E10 ultrasound machines (GE Healthcare Ultrasound, Milwaukee, WI, USA). Doppler recordings were performed using a 2.5‐ to 6‐MHz curvilinear transabdominal transducer. Color Doppler ultrasound was used to visualize the UA, and pulsed‐wave Doppler was then used to obtain the waveform from a free‐floating loop of the umbilical cord during fetal quiescence.^25^ The angle of insonation was kept below 15 degrees when possible. The high‐pass filter was set to the minimum, and a large sample volume (10–12 mm) was used for the pulsed Doppler recording. At least three uniform waveforms were obtained, and the recorded measurement reflected an average of three consecutive cardiac cycles. The UA‐PI was calculated as follows^26^: UA‐PI = ([peak systolic velocity] − [end diastolic velocity])/[time‐averaged maximum velocity]. The diagnosis of elevated UA‐PI (>95th centile) was based on the reference chart of Arduini and Rizzo.^12^

### 2.4 Definitions

Estimated fetal weight was calculated using the Hadlock formula,^27^ which incorporates all four biometric indices: biparietal diameter, head circumference, abdominal circumference, and femur length.

Small for gestational age was defined as EFW less than the 10th centile for gestational age according to the chart of Hadlock^28^ or birth weight below the 10th centile for gestational age based on a sex‐specific birth weight reference.^29^

Gestational age at the time of ultrasound examinations and birth was determined using the best obstetrical estimate, which included the date of in vitro fertilization (when applicable), first‐trimester ultrasound (based on Hadlock's crown‐rump length reference Table^30^), and the last menstrual period, in that order.^31^

### 2.5 Construction of UA‐PI Doppler reference

We compared two statistical approaches to construct the UA‐PI Doppler chart: a parametric method (Lambda‐Mu‐Sigma [LMS]) and a non‐parametric method (polynomial quantile regression).

The parametric LMS method^32^ uses three parameters Lambda (λ), Mu (μ), and Sigma (σ), which describe the skewness, central tendency, and variance of the distribution, respectively. Additional parameters may be estimated depending on the specific distributional family chosen. The analysis was performed using R version 4.4.1. The *lms* function from the *gamlss* package was used to automate the selection process for the appropriate LMS‐type centile estimation. For all subgroups, the optimal fit was achieved using the four‐parameter Box‐Cox t model. This model effectively captured data complexity by providing a robust framework that accounted for the central tendency, variability around the median, skewness, and tail heaviness. A significant advantage of such parametric approaches is that they allow for the calculation of exact *z*‐scores and centiles, which may be useful for both research and clinical purposes.

The second, non‐parametric approach was polynomial quantile regression,^18^, ^33^ which was used to estimate individual UA‐PI centiles. Cubic quantile regression was selected as it minimized the Akaike information criterion, and all three polynomial terms included in the model were statistically significant. This analysis was performed using the quantile regression function in the *gamlss* R package. The main advantage of such non‐parametric methods is their flexibility, as they do not assume a particular underlying distribution, making them suitable for modeling complex or irregular data patterns. However, limitations of non‐parametric methods include the inability to precisely calculate continuous centiles and the potential generation of less smooth curves, especially at the extremes of gestational age.

Analyses were performed at the examination level without explicitly modeling within‐pregnancy correlation. Given the small number of ultrasound examinations per pregnancy (one or two) and the large number of participants, this approach is common for cross‐sectional centile construction.^34^

### 2.6 Clinical characteristics affecting the UA‐PI Doppler reference

We explored the impact of the following clinical characteristics of the study population on the UA‐PI centile values: (1) fetal size—i.e. the minimum EFW centile cut‐off of the study population, using cut‐off values of more than the 10th, 25th, and 50th centiles and (2) parity, defined as nulliparous versus multiparous patients.

The rationale for considering EFW centiles is that pregnancies may be affected by FGR even when fetal weight is above the 10th centile.^4^, ^5^, ^6^ For example, a large population study found that the risk of stillbirth and other adverse perinatal outcomes begins to increase when birth weight falls below the 25th centile (rather than the 10th centile).^35^ Similarly, the rationale for considering parity is based on evidence showing an association between parity and improved placental development,^36^, ^37^, ^38^, ^39^ spiral artery remodeling,^40^ uterine artery Doppler,^41^, ^42^ and fetal growth.^43^

### 2.7 Statistical analyses

Standard descriptive statistics were used to summarize the baseline characteristics of the study population.

We explored the impact of the three factors described above—parity, minimal EFW centile inclusion cut‐off, and statistical method—on the UA‐PI centile values. McNemar test was used to compare the proportion of fetuses with UA‐PI greater than the 95th centile according to charts constructed from specific subgroups, with the corresponding proportion based on the chart constructed using the total cohort as the reference group.

We subsequently constructed and described a UA‐PI centiles chart based on the optimal combination of these factors, as determined by the exploratory analysis. The 50th and 95th centile curves of the new UA‐PI chart were compared with those of selected previously published UA‐PI Doppler charts. We also compared the proportion of ultrasound examinations in our cohort with abnormal UA‐PI values (>95th centile) according to each of these charts. Data were visualized and analyzed using Microsoft Excel (version 16.89.1), SPSS (version 29.0.2.0^20^), and GraphPad Prism (version 10.2.3 [347]). Statistical significance was set to *P* value less than 0.05.

## 3 RESULTS

### 3.1 Characteristics of the study cohort

**FIGURE 1:**
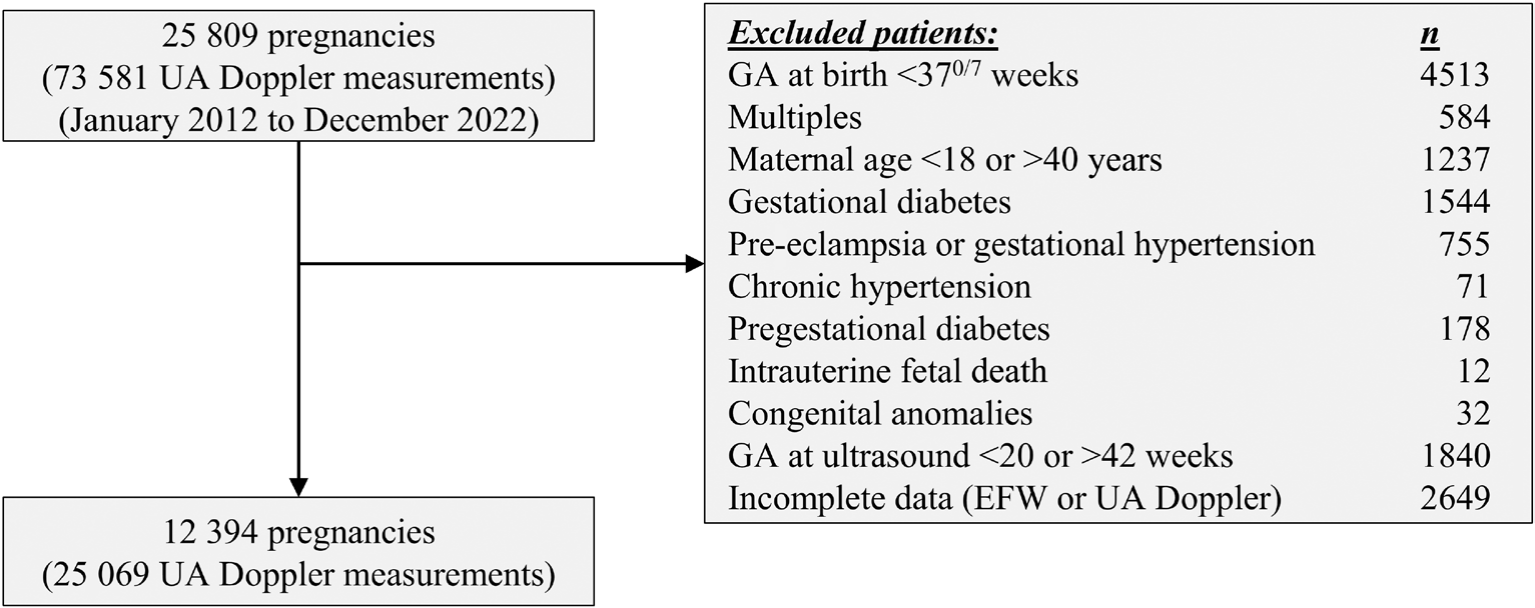
Selection of the study group. EFW, estimated fetal weight; GA, gestational age; UA, umbilical artery.

Overall, 25 809 patients underwent UA Doppler assessment during the study period. Of these, 12 394 patients, who underwent a total of 25 069 UA‐PI measurements, met the study criteria (Figure 1). The baseline characteristics of the study population are presented in Table 1. Approximately one‐third of the patients (35.6% [n=4,413]) were nulliparous. The proportions of infants with birth weights below the 10th and 3rd centiles for gestational age (12.1% [n=1,494] and 2.8% [n=355], respectively), along with the low rate of short‐term neonatal complications, confirm the low‐risk nature of the study population (Table 1).

**TABLE 1:** Characteristics and outcomes of the study cohort.^a^

Variable	Value
Number of patients (pregnancies)	12 394
Number of ultrasound examinations	25 069
Maternal characteristics and outcomes	
Maternal age, years	32.7 ± 4.1
Maternal age >35 years	3175 (25.6)
Nulliparity	4413 (35.6)
Cesarean delivery	4172 (33.7)
Gestational age at delivery, weeks	39.3 ± 1.2
Neonatal characteristics and outcomes	
Female sex	6162 (49.7)
Birth weight, g	3334 ± 463
Birth weight <10th centile^b^	1494 (12.1)
Birth weight <3rd centile^b^	355 (2.8)
5‐min Apgar <7	169 (1.4)
Arterial pH <7.1	52 (0.4)
Need for resuscitation	273 (2.2)
NICU admission	186 (1.5)

Abbreviations: NICU, neonatal intensive care unit.

^a^ Data are presented as mean ± standard deviation, number, or number (percentage).

^b^ Based on the Canadian sex‐specific birth weight chart of Kramer et al.^29^

### 3.2 Factors affecting the UA‐PI Doppler chart

We first explored the impact of the three selected methodologic factors that may contribute to the heterogeneity among published UA‐PI charts.

**FIGURE 2:**
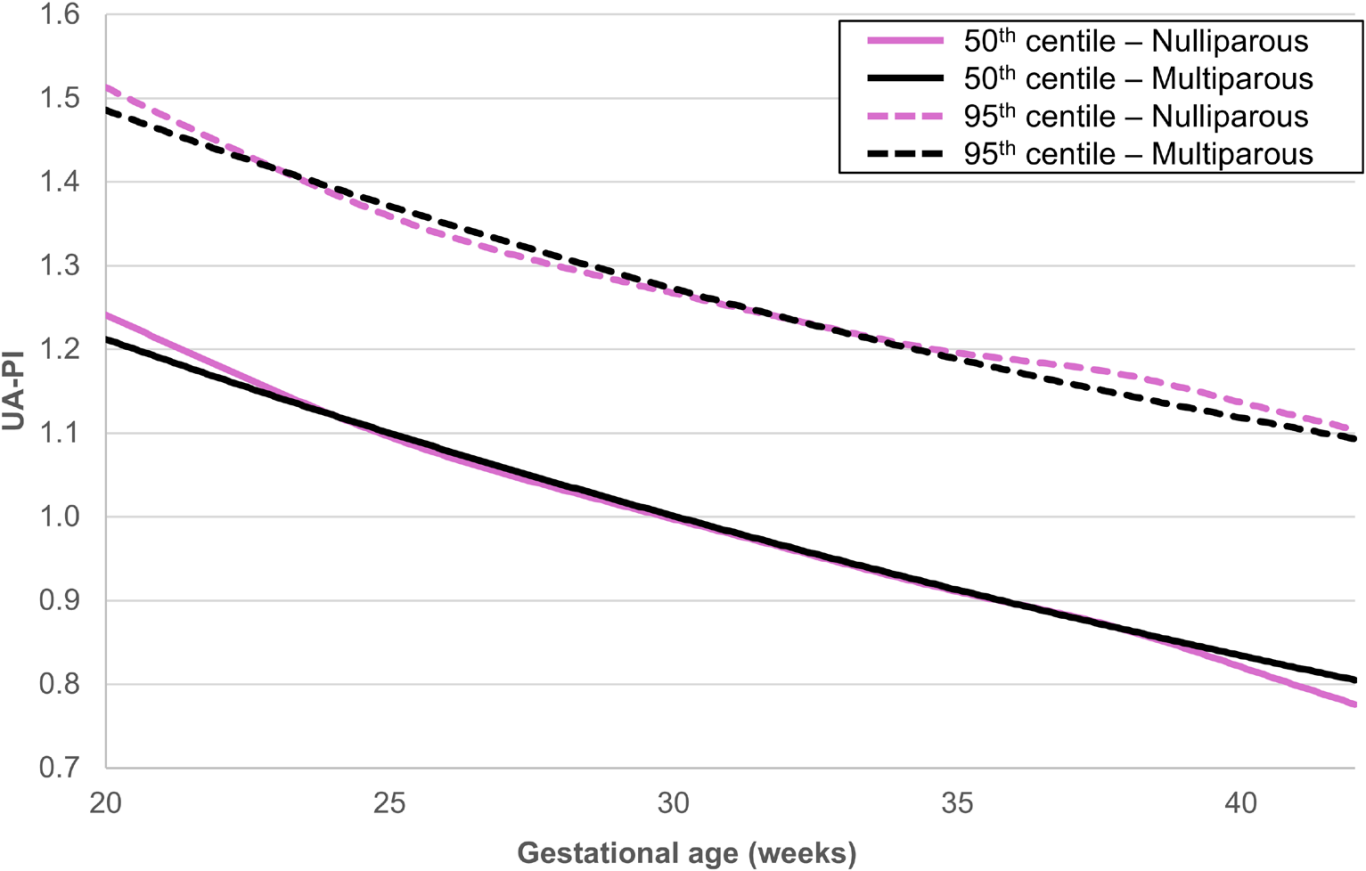
Umbilical artery pulsatility index (UA‐PI) reference charts based on parity. The 50th (solid lines) and 95th (dashed lines) centiles of UA‐PI were calculated for two subgroups based on parity: Nulliparous patients (pink lines) and multiparous patients (black lines). Centiles were estimated using the Lambda‐Mu‐Sigma method, with the cohort limited to fetuses with an estimated fetal weight (EFW) >10th centile.

When evaluating the effect of parity, we observed only minimal differences in UA‐PI 50th and 95th centiles between nulliparous and multiparous patients across the gestational age range (Figure 2). Consequently, the use of charts that are based on nulliparous versus multiparous patients had minimal impact on the proportion of ultrasound examinations with UA‐PI greater than the 95th centile, both in the total cohort (5.2% [n=1,300] versus 5.3% [n=1,317], respectively) and in the subgroup of SGA fetuses (10.4% [n=245] versus 10.6% [n=249], respectively) (Table 2).

**TABLE 2:** The effect of the study population and statistical method used to construct the umbilical artery pulsatility index (UA‐PI) reference chart on the proportion of ultrasound examinations with UA‐PI >95th centile.^a^

Methodologic factors used to construct the UA‐PI reference chart^b^	Proportion of examinations with UA‐PI >95th centile according to each of the charts			
	Within examinations of only SGA fetuses (*n* = 2348)	*P* value^c^	Within all ultrasound examinations (*n* = 25 069)	*P* value^c^
Overall cohort—reference (any parity, fetuses with EFW >10p, LMS method)	249 (10.6)	Reference	1313 (5.2)	Reference
By parity				
Only nulliparous patients	245 (10.4)	0.424	1300 (5.2)	0.283
Only multiparous patients	249 (10.6)	1.000	1317 (5.3)	0.481
By EFW centile				
Only fetuses with EFW >25th centile	269 (11.5)	<0.001	1475 (5.9)	<0.001
Only fetuses with EFW >50th centile	309 (13.2)	<0.001	1743 (7.0)	<0.001
By statistical method				
Quantile regression	255 (10.9)	0.031	1388 (5.5)	<0.001

Abbreviations: EFW, estimated fetal weight; LMS, Lambda‐Mu‐Sigma; SGA, small for gestational age; UA‐PI, umbilical artery pulsatility index.

^a^ Data are presented as number (percentage).

^b^ As a default, charts included fetuses with EFW >10th centile (except for the charts testing the effect of EFW centile) and using the LMS method (except for the charts testing the statistical method).

^c^ Refers to comparing the proportion of fetuses with UA‐PI >95th centile according to each reference chart with the corresponding proportion according to the reference chart constructed using the overall cohort of fetuses with EFW >10th centile and the LMS method.

**FIGURE 3:**
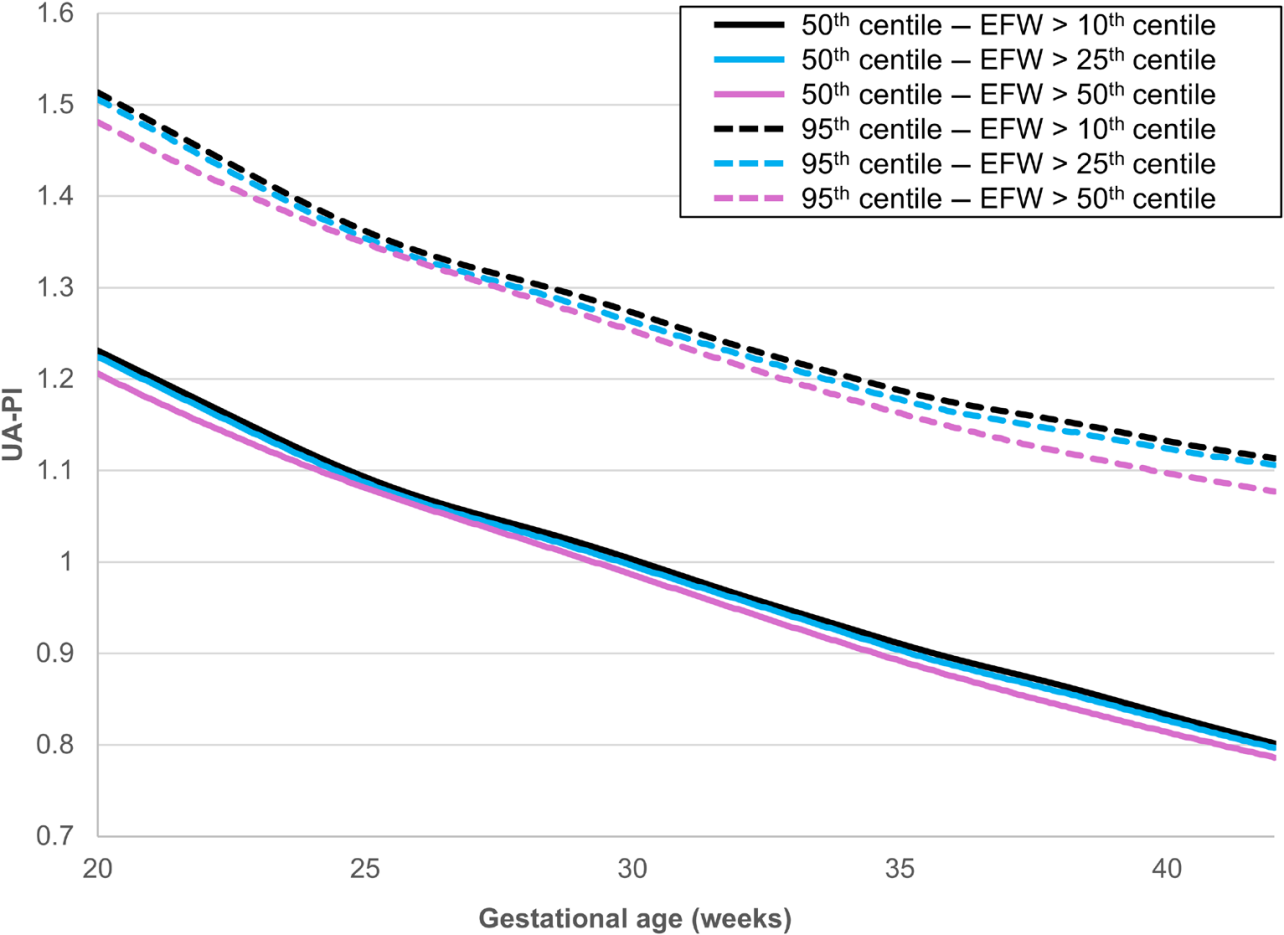
Umbilical artery pulsatility index (UA‐PI) reference charts based on estimated fetal weight (EFW) threshold. The 50th (solid lines) and 95th (dashed lines) centiles of UA‐PI were calculated for three subgroups based on EFW centiles: EFW >10th centile (black lines), >25th centile (blue lines) and >50th centile (pink lines). Centiles were estimated using the Lambda‐Mu‐Sigma method.

Next, we examined the effect of the study population's minimal EFW centile inclusion cut‐off (>10th, >25th, or >50th centile) on the UA‐PI centile values (Figure 3). The UA‐PI 50th and 95th centiles decreased as the minimal EFW centile inclusion cut‐off increased. The differences became more pronounced as pregnancy advanced and were especially noticeable after 35 weeks of gestation (Figure 3). Using charts constructed for fetuses with EFW greater than the 25th or 50th centiles was associated with a significantly higher proportion of ultrasound examinations with UA‐PI values greater than the 95th centile compared with the chart based on fetuses with EFW above the 10th centile, both in the total cohort (5.9% [n=1,475] and 7.0% [n=1,743] versus 5.2% [n=1,313], respectively, *P* < 0.001) and in the subgroup of SGA fetuses (11.5% [n=269] and 13.2% [n=309] versus 10.6% [n=249], respectively, *P* < 0.001) (Table 2).

**FIGURE 4:**
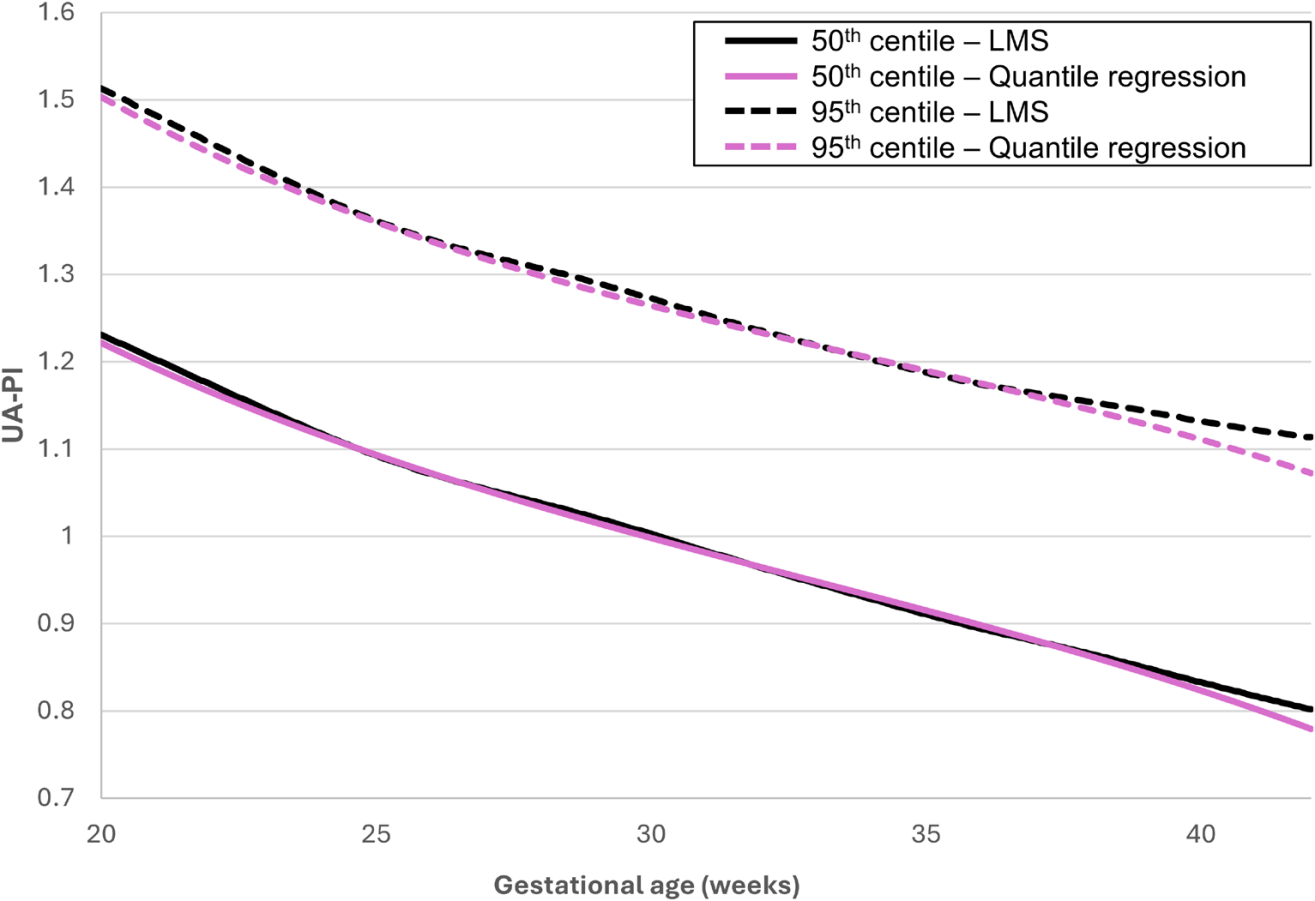
Umbilical artery pulsatility index (UA‐PI) reference charts based on the statistical approach. The 50th (solid lines) and 95th (dashed lines) centiles of UA‐PI were calculated in the overall cohort using two statistical approaches: Lambda‐Mu‐Sigma method (black lines) and quantile regression (pink lines). Centiles were estimated for the cohort of fetuses with an estimated fetal weight >10th centile.

Finally, we evaluated the impact of the statistical method on the UA‐PI centile values (Figure 4, Table 2). The LMS and quantile regression methods produced relatively similar UA‐PI centile charts, although the proportion of ultrasound examinations with UA‐PI greater than the 95th centile was slightly lower when using the LMS method, both in the total cohort (5.2% [n=1,313] versus 5.5% [n=1,388], *P* < 0.001) and in the subgroup of SGA fetuses (10.6% [n=249] versus 10.9% [n=255], *P* = 0.031) (Figure 4, Table 2).

### 3.3 Construction of a new UA‐PI Doppler reference

**FIGURE 5:**
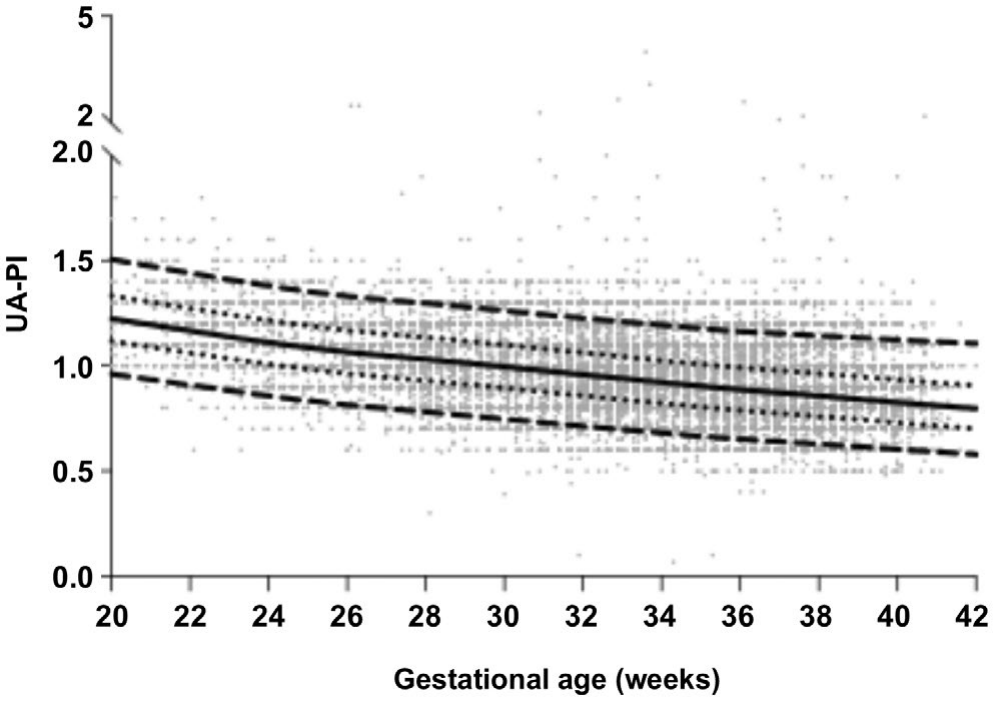
The newly constructed umbilical artery pulsatility index (UA‐PI) Doppler reference. Values were determined using fetuses with estimated fetal weight >25th centile, independent of parity, and using the Lambda‐Mu‐Sigma method. The 50th centile (solid line), 5th and 95th centiles (dashed lines), and 25th and 75th centiles (dotted lines) are shown. The centile curves are superimposed over the individual observations (gray circles).

Based on the findings described above, we selected an optimal UA‐PI centiles chart based on observations from all low‐risk pregnancy fetuses with EFW greater than the 25th centile, regardless of parity, using the LMS method. This decision was informed by the minimal effect of parity, existing evidence that the risk of stillbirth increases when fetal weight falls below the 25th centile,^35^ the marked increase in the proportion of ultrasound examinations with UA‐PI greater than the 95th centile when using a higher minimal EFW inclusion threshold of more than the 50th centile, and the advantages of the parametric LMS method (i.e. the ability to calculate exact UA‐PI centiles). The centiles of the newly constructed UA‐PI chart are presented in Table 3 and Figure 5.

**TABLE 3:** Umbilical artery pulsatility index Doppler centile values of the newly constructed reference chart.^a^

Gestational age, weeks	UA‐PI centiles						
	5th	10th	25th	50th	75th	90th	95th
20	0.962	1.021	1.118	1.224	1.334	1.439	1.506
21	0.935	0.994	1.09	1.196	1.304	1.408	1.474
22	0.909	0.967	1.062	1.167	1.274	1.377	1.442
23	0.883	0.941	1.035	1.139	1.245	1.346	1.411
24	0.858	0.915	1.009	1.112	1.217	1.317	1.381
25	0.835	0.892	0.985	1.087	1.192	1.291	1.354
26	0.815	0.872	0.964	1.066	1.17	1.269	1.332
27	0.797	0.854	0.947	1.048	1.152	1.251	1.314
28	0.781	0.838	0.93	1.032	1.136	1.235	1.298
29	0.764	0.821	0.913	1.014	1.118	1.218	1.281
30	0.747	0.803	0.895	0.996	1.1	1.199	1.263
31	0.73	0.785	0.876	0.977	1.081	1.181	1.245
32	0.713	0.768	0.858	0.959	1.063	1.163	1.227
33	0.697	0.751	0.841	0.94	1.045	1.145	1.211
34	0.681	0.735	0.823	0.922	1.026	1.128	1.194
35	0.666	0.718	0.805	0.904	1.008	1.111	1.178
36	0.652	0.703	0.789	0.887	0.992	1.096	1.164
37	0.64	0.69	0.774	0.872	0.978	1.084	1.154
38	0.628	0.677	0.76	0.858	0.964	1.072	1.144
39	0.616	0.664	0.746	0.843	0.95	1.06	1.134
40	0.603	0.65	0.73	0.827	0.935	1.047	1.124
41	0.591	0.636	0.716	0.811	0.92	1.035	1.115
42	0.579	0.623	0.701	0.797	0.906	1.024	1.106

Abbreviation: UA‐PI, umbilical artery pulsatility index.

^a^ Values were determined using fetuses with estimated fetal weight >25th centile, irrespective of parity, and using the Lambda‐Mu‐Sigma method.

### 3.4 Comparison with previously published UA‐PI charts

**FIGURE 6:**
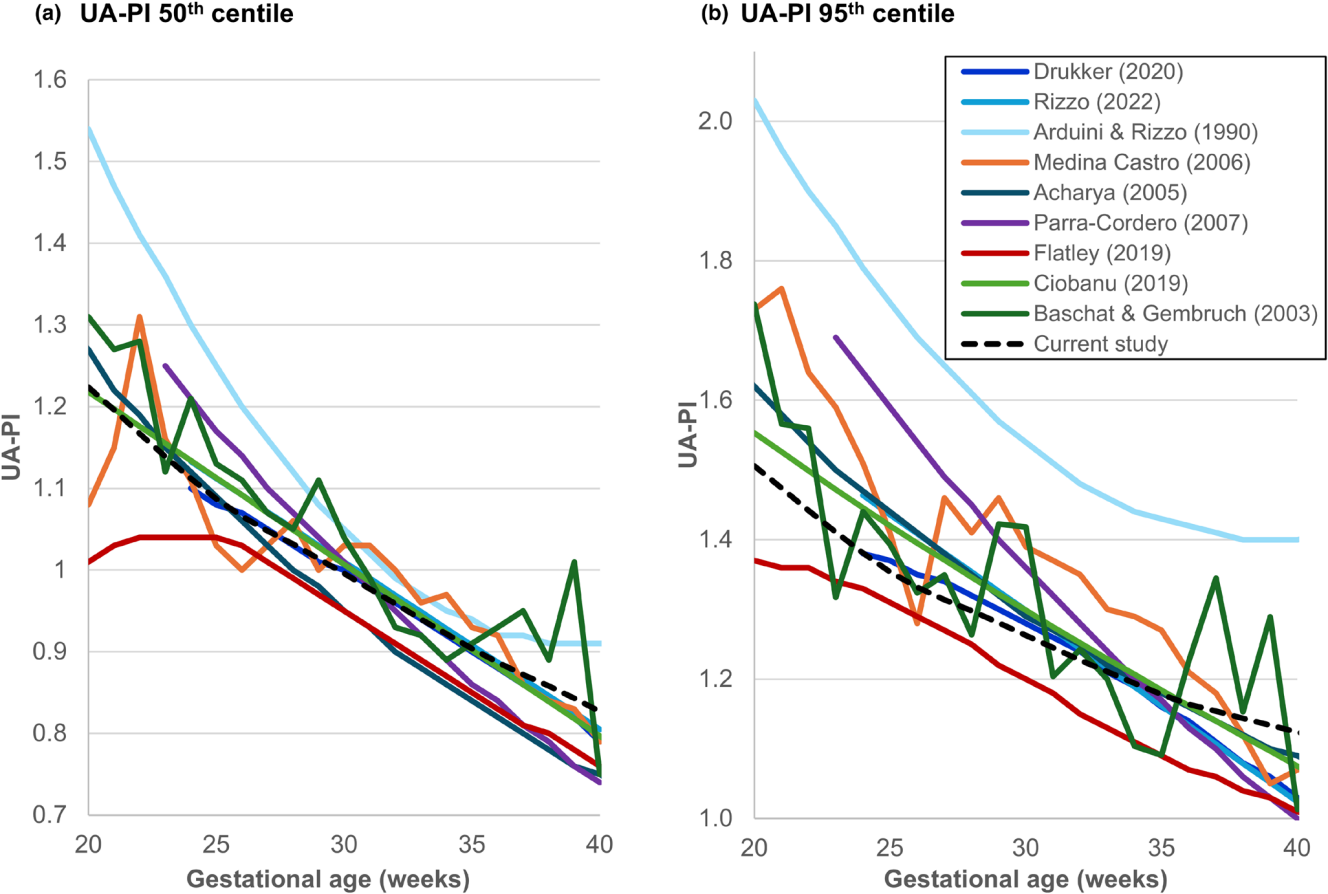
Comparing the newly constructed umbilical artery pulsatility index (UA‐PI) reference chart to existing UA‐PI references. The 50th (a) and 95th (b) centiles of the newly constructed UA‐PI chart (dashed black line) are compared with the color‐coded corresponding centiles of previously published charts.

**FIGURE 7:**
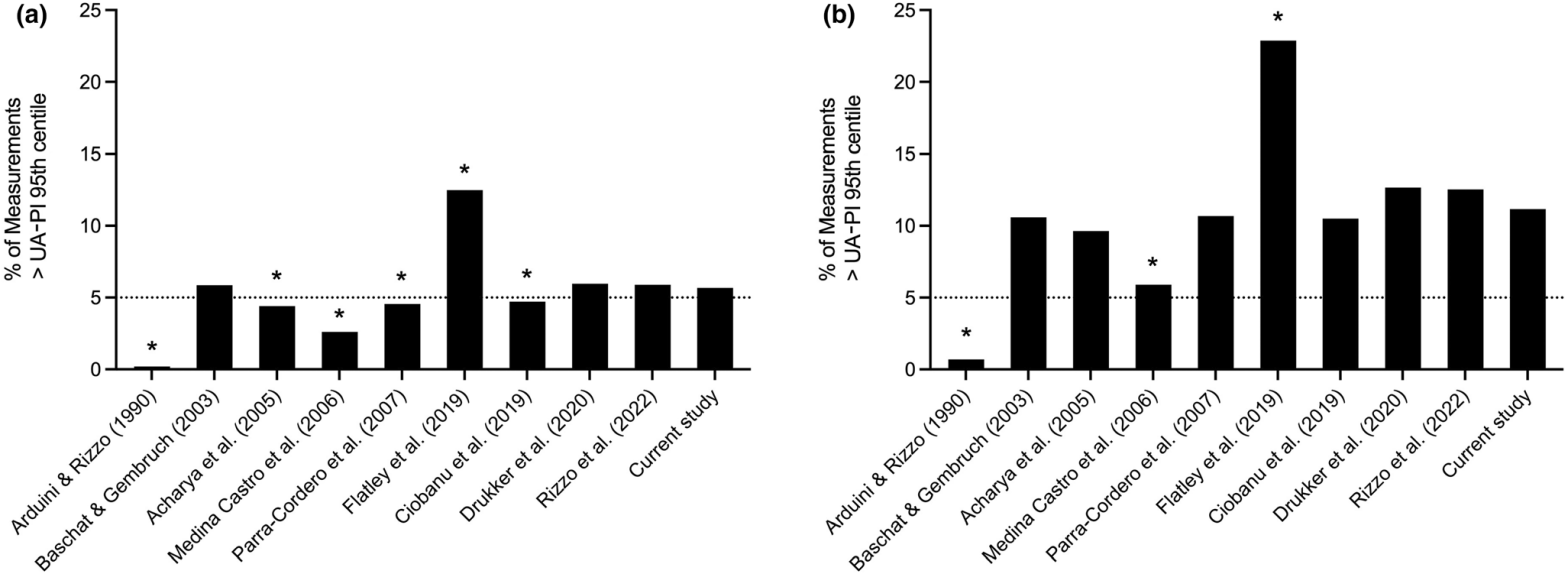
Effect of chart selection on the proportion of fetuses with umbilical artery pulsatility index (UA‐PI) >95th centile. The proportion of measurements in our study cohort with UA‐PI >95th centile according to each reference chart is shown for the overall cohort (a) and the subgroup of small‐for‐gestational‐age fetuses (b). *Proportion is significantly different (*P* < 0.05) compared with the proportion based on the chart constructed in the current study.

The 50th and 95th centiles of the newly constructed UA‐PI chart were compared with the corresponding centiles from commonly used published charts (Figure 6). Overall, we observed substantial heterogeneity among the charts, with the proportion of fetuses in our cohort diagnosed with UA‐PI greater than the 95th centile ranging from 0.2% to 12.5% in the overall cohort and from 0.7% to 22.9% in the subgroup of SGA fetuses (Figure 7). The reference chart by Arduini and Rizzo^12^ had the highest values for the 50th and especially the 95th centiles, resulting in the lowest proportion of fetuses classified with UA‐PI greater than the 95th centile (Figure 7). In contrast, the chart by Flatley et al.^16^ had the lowest 50th and 95th centile values, yielding the highest proportion of fetuses with UA‐PI greater than the 95th centile (Figure 7). The charts by Medina‐Castro et al.^19^ and Baschat & Gembruch^27^ were notably jagged and irregular in shape (Figure 6).

Overall, the UA‐PI 95th centile of our newly constructed chart was most similar to those of the more recent charts by Drukker et al.^17^ and Rizzo et al.,^24^ as reflected by their similar centile curve (Figure 6b) and the comparable proportion of fetuses with UA‐PI greater than the 95th centile in both the total cohort and the subgroup of SGA fetuses (Figure 7a,b, respectively).

## 4 DISCUSSION

In the current study, we explored and demonstrated the potential impact of selected factors on UA‐PI centile values using a large, contemporary cohort of healthy, uncomplicated singleton pregnancies. We identified the following key findings: (1) parity had minimal impact on UA‐PI centile values; (2) the UA Doppler centile values decreased progressively with increasing minimal EFW centile cut‐off of the population used to construct the chart; (3) the parametric LMS method and non‐parametric quantile regression yielded relatively similar UA‐PI centile values; and (4) we constructed a new UA‐PI centiles chart using the LMS method based on fetuses with EFW greater than the 25th centile independent of parity; when compared with previously published charts, this new reference showed high similarity to two more recent charts, but notable discrepancies in the UA‐PI 95th centiles when compared with several other published references.

Accurate diagnosis of FGR—that reliably distinguishes growth‐restricted fetuses from those who are constitutionally SGA—is crucial to avoid unnecessary use of resources, potentially harmful interventions, and parental anxiety when SGA is misclassified as FGR.^5^ UA Doppler is a key tool for the diagnosis and management of early‐onset FGR, and its use in clinical decision making has been shown to reduce the risk of perinatal death and the rate of obstetrical interventions in clinically high‐risk pregnancies.^44^ The increased UA Doppler impedance observed in cases of FGR is believed to reflect reduced placental villous volume, diminished placental surface area, and impaired branching of the terminal villi. These pathologic changes mostly arise from impaired trophoblastic invasion and abnormal spiral artery remodeling, resulting in reduced placental perfusion and maternal vascular malperfusion (MVM) as observed on placental pathology.^45^, ^46^ However, the utility of UA Doppler in accurately diagnosing FGR due to placental MVM pathology in clinical practice^47^ is limited by the heterogeneity of existing UA Doppler reference charts [12–20]. In the current study, we explored the potential contribution of several methodologic factors to this heterogeneity.

The rationale for exploring the impact of parity on UA‐PI values is its association with improved placental development,^36^, ^37^, ^38^, ^39^ spiral artery remodeling,^40^ uterine artery Doppler indices,^41^, ^42^ and fetal growth.^43^ However, in our population of low‐risk uncomplicated pregnancies, we found that parity had only a minimal effect on the UA‐PI centile values, suggesting that variation in the relative proportion of nulliparous versus multiparous individuals across studies is unlikely to explain the heterogeneity among published UA‐PI reference charts. In contrast to our findings, Ciobanu et al.^15^ reported that the median UA‐PI values were significantly lower in multiparous than in nulliparous women. However, it should be noted that unlike our study, which included low‐risk uncomplicated pregnancies, their cohort was based on an unselected population. As such, their observed relationship between parity and UA‐PI values could have been confounded by clinical factors such as pre‐eclampsia and abnormal placentation, both of which are strongly associated with nulliparity.^48^

The second factor we considered was the minimal EFW centile cut‐off used to define the cohort for constructing the UA‐PI centiles chart. The rationale for exploring this factor is that pregnancies can be affected by placental dysfunction and FGR (and therefore exhibit pathologic UA‐PI values) even when fetal weight or birth weight is above the 10th centile.^4^, ^5^, ^6^, ^9^, ^35^ In addition, there is evidence suggesting a direct correlation between UA‐PI values and fetal size. Several studies found that large‐for‐gestational age fetuses (birth weight >90th centile) and those with birth weight greater than 4000 g have lower UA‐PI values than both appropriate for gestational age and SGA fetuses,^22^, ^49^, ^50^, ^51^, ^52^ a finding attributed to the positive correlation between fetal size and both placental size and umbilical cord diameter.^53^, ^54^, ^55^, ^56^, ^57^ Indeed, we found that UA Doppler centile values decreased progressively as the minimal EFW centile inclusion cut‐off increased. This finding suggests that variability in the distribution of EFW centiles across study cohorts may have contributed to the heterogeneity observed among published UA‐PI charts. Our finding is consistent with that of Acharya et al.,^13^ who reported a negative correlation between UA‐PI values and both birth weight and placental weight in a longitudinal study of 130 low‐risk singleton pregnancies. Taking our findings into account, along with previous evidence identifying the 25th centile birth weight cut‐off as a threshold associated with increased risk of stillbirth,^35^ we chose to construct our UA‐PI centiles chart using pregnancies with EFW greater than the 25th centile. We considered this cut‐off to provide an optimal balance between the risk of including pregnancies affected by FGR (which could falsely elevate UA‐PI values) and restricting the cohort to larger‐than‐average fetuses (which could falsely lower UA‐PI values).

The third factor we considered was the statistical method used to construct the UA‐PI chart. Most published UA‐PI charts have been constructed using parametric methods, which model both the central tendency (e.g. mean) and variance as functions of gestational age, thereby allowing the calculation of exact UA‐PI centiles.^13^, ^14^, ^15^, ^16^, ^17^ Others have used non‐parametric approaches, such as quantile regression, arguing that these methods offer a more flexible means of estimating gestational age‐dependent variables without the need to rely on assumptions regarding UA‐PI distribution at each gestational age.^18^, ^24^ However, non‐parametric approaches estimate the UA‐PI values only at specific, predetermined centiles during gestation and do not permit the calculation of exact continuous UA‐PI centiles for a given UA‐PI measurement at a specific gestational age.^58^ In our study, we found that both methods yielded relatively similar UA‐PI 50th and 95th centile values, suggesting that the choice of the statistical method is unlikely to be a major contributor to the heterogeneity observed among published UA‐PI charts. For the construction of our UA‐PI centiles chart, we chose to use the parametric LMS method because of several key advantages: (1) it models the distribution of UA‐PI across gestation using a formula that can be easily integrated into ultrasound machines or reporting software, thereby enhancing its clinical usability; (2) the ability to determine exact UA‐PI centiles may be valuable for research purposes, such as identifying optimal diagnostic thresholds; and (3) future multimodal prediction and diagnostic models for FGR are likely to incorporate variables in a continuous fashion (e.g. exact EFW centile and UA‐PI centile) rather than dichotomously.^59^

We identified considerable heterogeneity in the UA‐PI 95th centile values among published charts, which in turn has a substantial impact on the proportion of SGA fetuses diagnosed with FGR.^20^, ^21^ This finding emphasizes the need to identify the optimal UA‐PI chart for clinical practice as part of broader efforts to standardize the diagnosis of FGR.^60^ However, none of the current guidelines on FGR or obstetrical ultrasound provide a recommendation regarding which UA chart should be used.^5^, ^6^, ^7^, ^8^, ^9^, ^10^, ^11^, ^25^ Moreover, the persistence of heterogeneity among UA‐PI charts even when only studies with adequate methodology are considered^20^, ^21^ suggests that refining the methodologic aspects alone may not be sufficient to resolve this issue. An alternative approach is the outcome‐based comparison, where charts are evaluated based on their diagnostic accuracy for placenta‐mediated FGR. A recent study comparing four UA‐PI charts found that all four performed equally poorly in predicting SGA at birth and adverse perinatal and obstetrical outcomes, with area under the receiver operating characteristics curve values of 0.55–0.60.^61^ This finding highlights the importance of choosing appropriate outcomes when comparing UA‐PI charts. Adverse perinatal outcomes are often primarily driven by prematurity and are not necessarily specific indicators of placenta‐mediated FGR. Furthermore, because UA‐PI is typically measured when fetal weight is below the 10th centile, SGA at birth is unlikely to serve as an informative or specific outcome. Instead, we propose that more appropriate outcomes for evaluating UA‐PI charts include the progression to late Doppler abnormalities (such as absent or reversed end‐diastolic flow in the UA or abnormal ductus venosus Doppler) or abnormal placental pathology findings, both of which provide a more definitive and gestational age‐independent indication of placental insufficiency.

The main strengths of the current study are the large sample size and the strict exclusion criteria, which ensured that the study cohort represents low‐risk singleton pregnancies unlikely to be affected by placental insufficiency. This contrasts with studies that included complicated or unselected pregnancies, where the resulting UA‐PI chart may be variably influenced by the underlying rate of placental dysfunction within each study cohort, thereby potentially limiting the external validity of the derived charts. Another strength is that, in our settings, UA Doppler measurements are routinely acquired in all patients referred for fetal growth or biophysical profile assessment, regardless of fetal size, minimizing the risk of selection bias. Finally, to the best of our knowledge, this is the first study to systematically explore and illustrate the impact of study population characteristics (parity and fetal size) and statistical methods on UA‐PI centiles chart development.

The current study has several limitations. First, because of its retrospective design, we were unable to blind the sonographers during data acquisition or report quality control measures. Additionally, because of the retrospective nature of the study, information on other potentially important pregnancy characteristics (such as maternal body mass index and racial origin) was not available, limiting our ability to evaluate the influence of these factors on UA‐PI centiles. Second, our approach may introduce some circularity, as excluding fetuses with low EFW to construct the UA‐PI chart inevitably lowers centile values and increases the likelihood that smaller fetuses are classified as abnormal. Nevertheless, the inverse association between UA‐PI and fetal size likely reflects, at least in part, underlying placental insufficiency rather than fetal size per se. Third, this was a single‐center study without external validation, which may limit the generalizability of our findings.

In summary, we identified that variation in the distribution of EFW centiles among populations used to construct UA‐PI reference charts—but not differences in parity or statistical methodology—may contribute to the heterogeneity observed among published UA‐PI charts. Future studies aiming to construct improved UA Doppler charts should be aware of this finding and carefully consider the EFW centile inclusion cut‐off for their study cohorts. International standardization of UA‐PI interpretation is an important goal that may be enhanced by these observations.^62^

## AUTHOR CONTRIBUTIONS

NM and SR contributed to the conception, design, analysis and interpretation of the data, and drafting of the manuscript. JCK, AV, and VS contributed to the interpretation of the data and revising of the manuscript. All authors approved the final version of this manuscript as submitted, and all authors agree to be accountable for all aspects of the work in ensuring that questions related to its accuracy or integrity are appropriately resolved.

## FUNDING INFORMATION

Dr. Melamed holds the Waugh Family Chair in Twin Fetal Medicine Research at the Sunnybrook Health Sciences Centre and the University of Toronto.

## CONFLICT OF INTEREST STATEMENT

The authors have no conflicts of interest.

## Data Availability

The data that support the findings of this study are available on request from the corresponding author. The data are not publicly available due to privacy or ethical restrictions.
